# Alzheimer's disease-associated inflammatory pathways might contribute to osteoporosis through the interaction between *PROK2* and *CSF3*

**DOI:** 10.3389/fneur.2022.990779

**Published:** 2022-09-20

**Authors:** Wenzheng Zhang, Ya Zhang, Naixia Hu, Anying Wang

**Affiliations:** ^1^Department of Joint Sports Medicine, The Affiliated Taian City Central Hospital of Qingdao University, Taian, China; ^2^Department of Pathology, The Second Affiliated Hospital of Shandong First Medical University, Taian, China; ^3^Neurointensive Care Unit, The Second Affiliated Hospital of Shandong First Medical University, Taian, China; ^4^Department of Orthopedics, The Second Affiliated Hospital of Shandong First Medical University, Taian, China

**Keywords:** Alzheimer's disease, osteoporosis, *PROK2*, *CSF3*, bio-informatics analysis, biomarkers, neurovascular

## Abstract

This study aimed to explore the potential molecular pathways and targets of Alzheimer's disease leading to osteoporosis using bioinformatics tools. The Alzheimer's and osteoporosis microarray gene expression data were retrieved from the Gene Expression Omnibus, and differentially expressed genes in the blood microenvironment related to Alzheimer's disease and osteoporosis were identified. The intersection of the three datasets (GSE97760, GSE168813, and GSE62402) was used to obtain 21 co-expressed targets in the peripheral blood samples in patients with Alzheimer's disease and osteoporosis. Based on the degree algorithm, the top 10 potential core target genes related to these diseases were identified, which included *CLEC4D, PROK2, SIGLEC7, PDGFB, PTCRA, ECH1*, etc. Two differentially expressed mRNAs, Prokineticin 2 (*PROK2*) and three colony-stimulating factor 3 (*CSF3*), were screened in the GSE62402 dataset associated with osteoporosis. Protein–protein rigid docking with ZDOCK revealed that *PROK2* and *CSF3* could form a stable protein docking model. The interaction of *PROK2* and *CSF3*, core genes related to osteoporosis inflammation, plays an important role in the mechanism of osteoporosis in patients with Alzheimer's. Therefore, abnormalities or alterations in the inflammatory pathways in the peripheral blood samples of Alzheimer's patients may affect the course of osteoporosis.

## Introduction

Osteoporosis (OP) is a bone metabolic disease that is common and highly prevalent in the elderly population (1). The prevalence of OP in the elderly increases with age (2). OP reduces bone strength and increases the risk of fracture in these patients (3, 4). Globally, osteoporotic fractures are an economic burden on society and the patients' families. They are also associated with high disability and mortality rates in elderly patients, which raise serious concerns about their health in today's aging society (5–8).

Alzheimer's disease (AD), which is yet another concern in the elderly, has been found to have a high incidence in the elderly along with OP. Previous studies have shown a prevalence of osteoporosis of 27% in patients with Alzheimer's disease, compared to 16% in residents without dementia (9). Beta-amyloid, *APOE4*, vitamin K, and vitamin D may be important proteins that interconnect AD and OP (10). Vitamin deficiency significantly increases AD risk. Interestingly, vitamin levels within a certain range positively correlate with cognitive performance (11–13). Also, alterations in vitamin D levels in the serum in middle-aged and elderly populations are associated with decreased bone mineral density (14). The AD mouse model, APP/PS1 transgenic mice, had significantly different bone microarchitecture and bone density parameters compared to wild-type mice and was more susceptible to OP (15). *In vitro* and *in vivo* studies in AD transgenic mouse models showed an enhanced amyloid beta (Aβ) peptide expression in bone tissue. Furthermore, an increase in Aβ peptide levels induces changes in bone mineral density, affecting the balance between bone formation and bone resorption, leading to OP (16). In addition, the Wnt/β-catenin signaling pathway plays an important role in AD and OP pathogenesis due to its role in inflammation (17, 18). Therefore, it is tempting to postulate a correlation between AD and OP. Patients with AD are cognitively impaired and prone to physical injuries. It is important to understand how AD regulates OP in patients to prevent OP occurrence and its treatment in patients with AD.

With the advancement in bioinformatics and high-throughput sequencing, it is now possible to screen the differentially expressed genes (DEGs) using microarray gene expression profiling (19–23). Publicly available databases and repositories that store information on gene expression, microarrays, and clinical samples can help understand the underlying mechanism of the disease and screen potential molecular targets quickly and efficiently before their use in clinical settings (24–26). This study aimed to investigate the molecular mechanism of OP in patients with AD using data retrieved from the Gene Expression Omnibus (GEO) database. The potential molecular pathways and biological processes associated with OP in patients with AD were explored using bioinformatics tools. Finally, we identified key targets for preventing and treating OP in patients with AD. This will provide valuable insights into understanding the pathogenesis and progression of OP in patients with AD.

## Materials and methods

### Target gene identification

The gene expression microarray data on “Alzheimer's disease” and “osteoporosis” were retrieved from the GEO database (https://www.ncbi.nlm.nih.gov/geo/). The data were screened using the following criteria: (i) keywords “Alzheimer's disease,” “osteoporosis,” (ii) peripheral blood, and (iii) human. The expression matrix data were corrected and normalized using the Bioconductor R package (R version 4.0.4). The differentially expressed mRNAs in the peripheral blood samples of patients with “AD” and “OP,” that is, the differentially expressed genes (DEGs) associated with AD and OP, were found in compared to normal healthy adults. The DEGs between the two groups were calculated using the linear models for the microarray data (limma package), with the screening criteria of *P* < 0.05 and absolute value of fold change ≥ 1.41 (|log2 FC| ≥ 0.50). We used the statistical tests built into the ggpubr package for statistical testing.

### Screening and co-expression of differential genes in AD and OP and PPI network construction

Using the Venn R package, Venn graphs were created by intersecting AD-related and OP-related DEGs. The Search Tool for the Retrieval of Interacting Genes/Proteins database (https://string-db.org/) was used to construct the protein–protein interaction (PPI) network and generate PPI relationship data. The PPI network model was further visualized by Cytoscape 3.7.2. The PPI network was topologically analyzed according to degree values to screen for the core target proteins.

### Gene ontology functional analysis and KEGG pathway enrichment

The clusterProfiler, an R package, was used to perform Gene Ontology (GO) and Kyoto Encyclopedia of Genes and Genomes (KEGG) enrichment analysis on AD-OP-related DEGs. The species was set to human for this analysis. The signaling pathways were mapped using the “Pathview: an R/Bioconductor package.”

### Establishment of OP-inflammatory-related gene expression matrix

The gene expression matrix of the OP transcriptome profile was established with the inflammatory response-related genes extracted from the Gene Set Enrichment Analysis (GSEA) database as previous researches (27–29). The differentially expressed mRNAs between the OP group and normal healthy groups were calculated using the limma package. The “heatmap” package was used to construct maps of gene expression and cluster the DEGs.

### Relative expression of core target genes

The microarray data of the OP-related gene expression matrix were retrieved from GEO, and the expression of core DEGs in each sample was derived based on the core targets obtained from the pre-screening. The “ggpubr package” was used to analyze the relative expression of the core targets in the OP expression data. *P* < 0.05 was considered statistically significant. A box plot of the relative expression of the core targets was plotted (R version 4.0.4).

### GO and KEGG enrichment analysis of OP-inflammation-related genes

GO and KEGG pathway enrichment analysis of OP-inflammation-related genes were done using the Scatterplot3d: 3D graphics, clusterProfiler in R package software, and Perl software package.

### Molecular docking to validate the interactions between inflammatory proteins

Rigid protein–protein docking (ZDOCK) was performed between inflammatory proteins to study the reciprocal relationships. The PDB format of the protein structural domain was downloaded from the Protein Data Bank PDB database (http://www.rcsb.org/). The protein structure was imported into Discovery Studio 2019 software to dehydrate and dephosphorylate the proteins. The upstream protein of the inflammatory pathway was set as the receptor protein, and the downstream protein was selected as the ligand–protein. The angular step size was set to 15°. The ZDOCK module was run to identify the docking site and calculate the ZDOCK Score. When molecular dynamics simulation (MDS) finds the docking site, the two form a stable docking (30–33). The results of protein–protein molecular docking are shown in 2D format.

## Results

### Screening for disease targets

**Figure 1:**
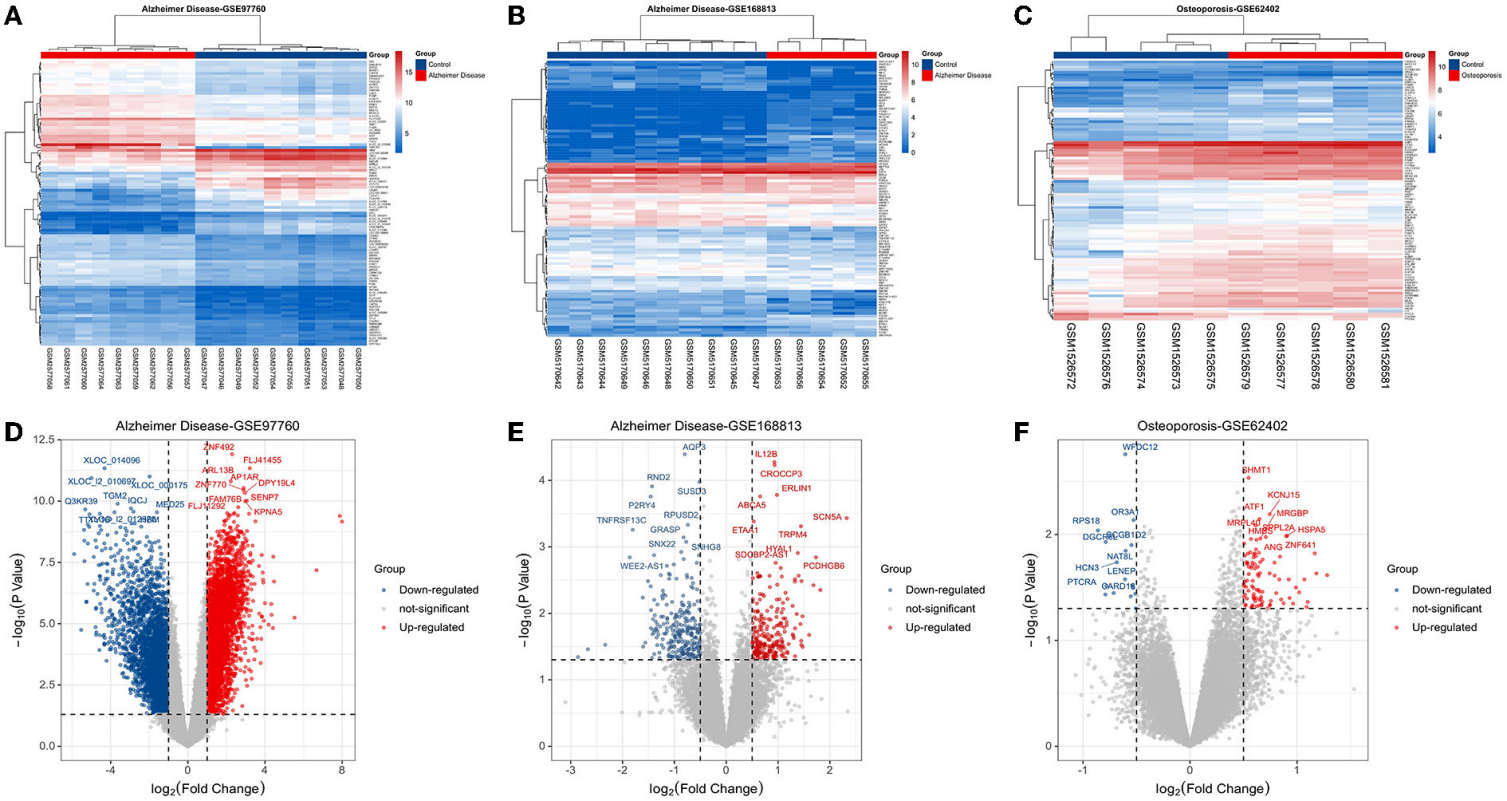
Disease target screening. **(A)** Heat map of differentially expressed genes in GSE97760; **(B)** heat map of differentially expressed genes in GSE168813; **(C)** heat map of differentially expressed genes in GSE62402; **(D)** volcano map of differentially expressed genes in GSE97760; **(E)** volcano map of differentially expressed genes in GSE168813; **(F)** volcano map of differentially expressed genes in GSE62402.

Based on the keywords used and screening criteria set, nine patients with AD and 10 normal healthy individuals from the GSE97760 dataset retrieved from GEO were included in the study. A total of 7,370 differentially expressed mRNAs, of which 4,003 upregulated and 3,367 downregulated mRNAs, were obtained. In the GSE168813 dataset, five patients with AD and 10 normal healthy individuals were included in the study. In this dataset, 499 differentially expressed mRNAs were identified, of which 236 mRNAs were upregulated, and 263 mRNAs were downregulated. In the GSE62402 dataset, five OP patients and five normal healthy individuals were included in the study, and 110 differentially expressed mRNAs (94 upregulated and 16 downregulated mRNAs) were obtained. The heat map generated is shown in Figures 1A–C. The transcriptome differential expression data were represented by constructing a volcano map, as shown in Figures 1D–F.

### Detection of AD-OP-related DEGs in peripheral blood and construction of PPI networks

**Figure 2:**
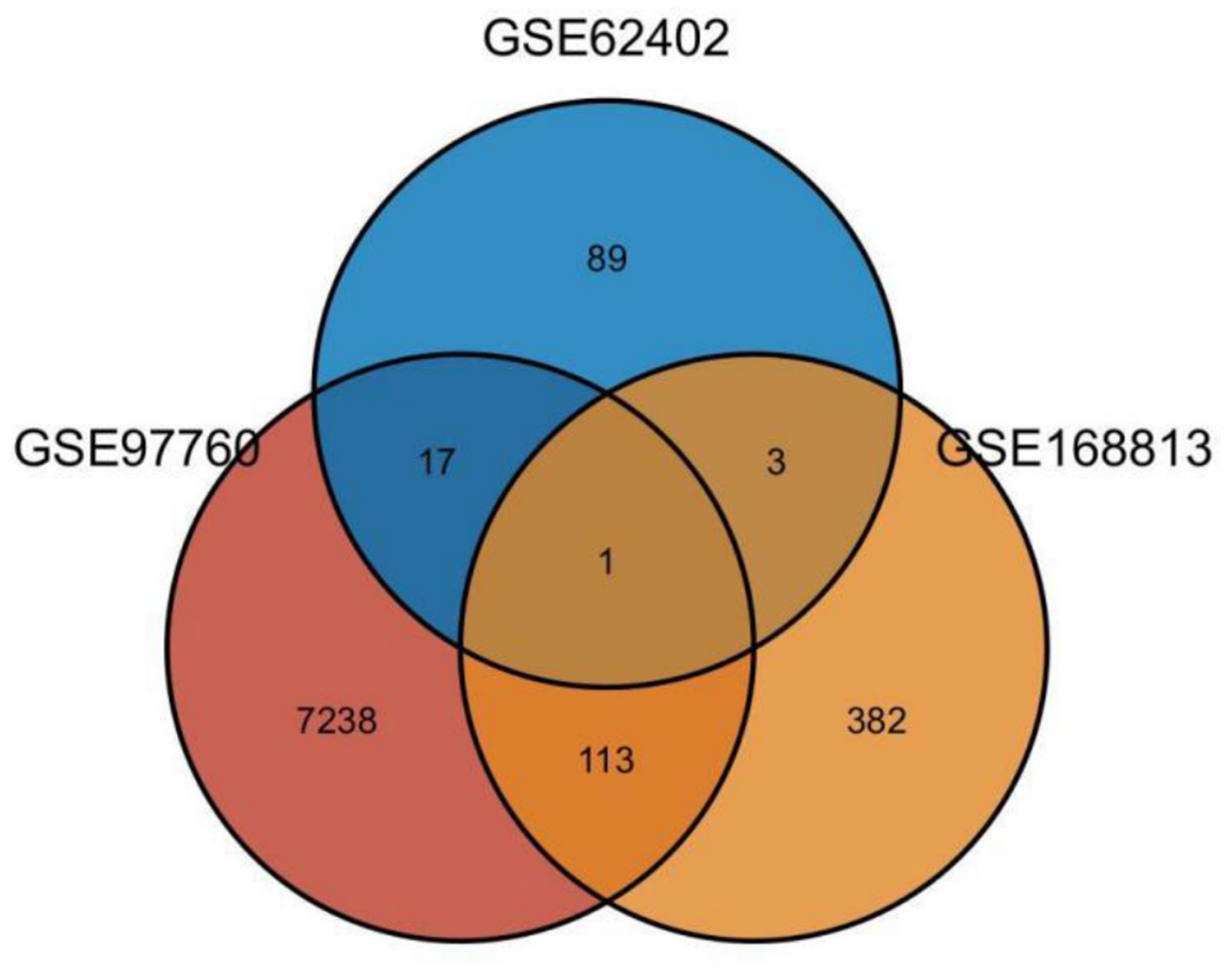
Venn diagram of differential co-expressed genes in Alzheimer's disease and osteoporosis in the blood microenvironment.

**Figure 3:**
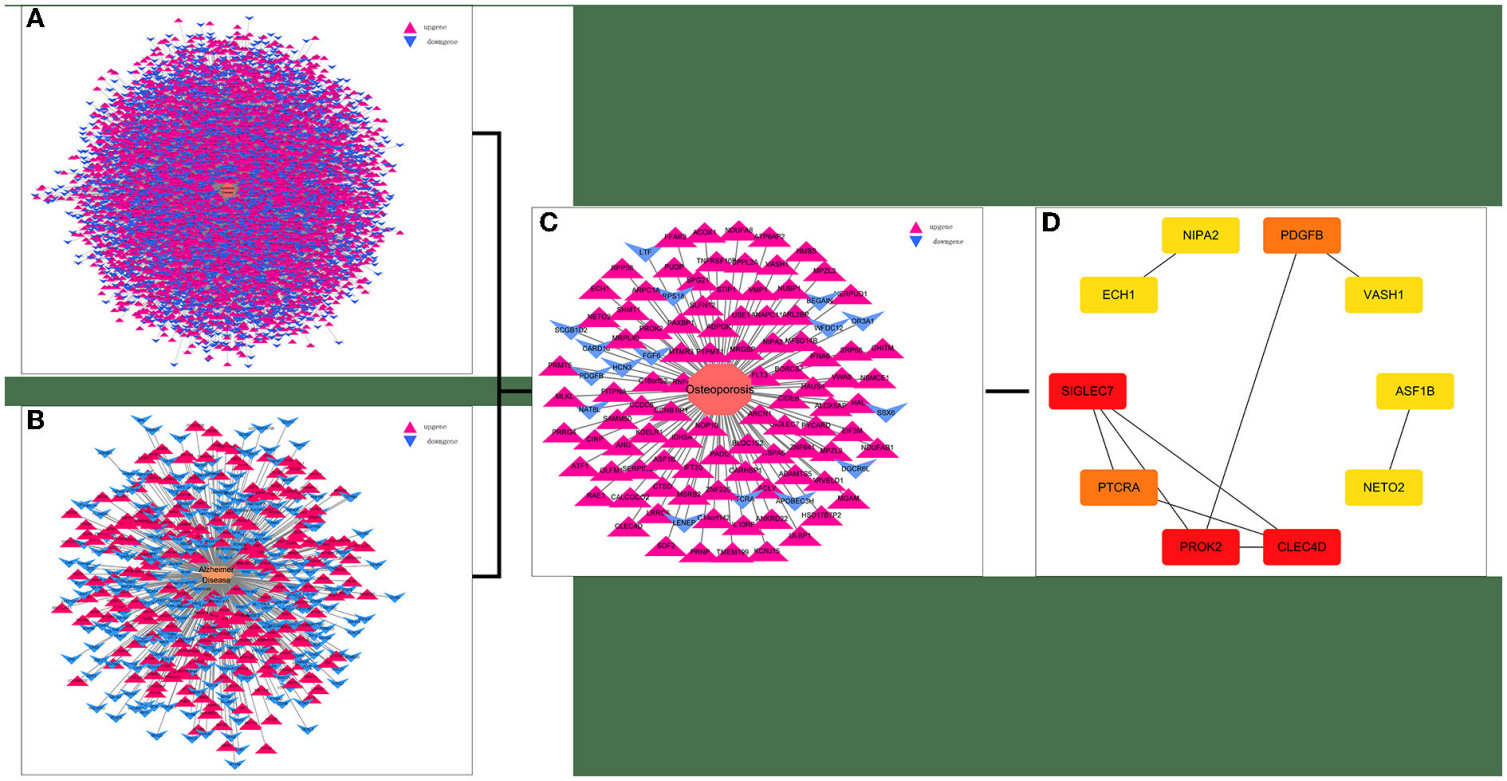
Screening for AD-OP-related DEGs in peripheral blood and construction of PPI networks. **(A,B)** GSE168813 differentially expressed gene interaction network; **(C)** GSE62402 differentially expressed gene interaction network; **(D)** top 10 potential core target genes based on degree values.

Twenty-one AD-OP-related DEGs were obtained from the intersection of the DEGs of the three microarray datasets (Figure 2). The AD-OP-target gene network was constructed by Cytoscape software (Figures 3A–C). Protein–protein interaction of the AD-OP-related DEGs was constructed using Cytoscape software. The top 10 potential core target proteins (Figure 3D), including *CLEC4D, PROK2, SIGLEC7, PDGFB, PTCRA*, and *ECH1*, were obtained using the CytoHubba plugin in Cytoscape software based on degree size screening (34).

### Results of the GO and KEGG enrichment analysis

**Figure 4:**
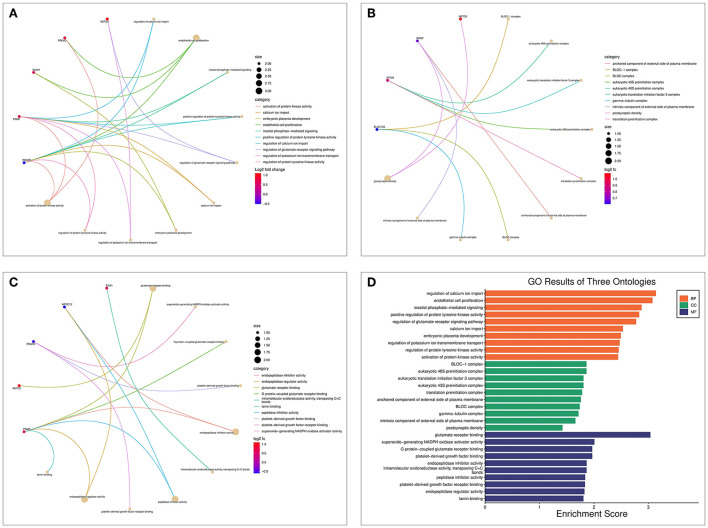
Results of the GO enrichment analysis for AD-OP-related DEGs. **(A)** Chord diagram of biological processes functional analysis; **(B)** chord diagram of cell component functional analysis; **(C)** chord diagram of molecular function functional analysis; **(D)** histogram of GO enrichment analysis.

**Figure 5:**
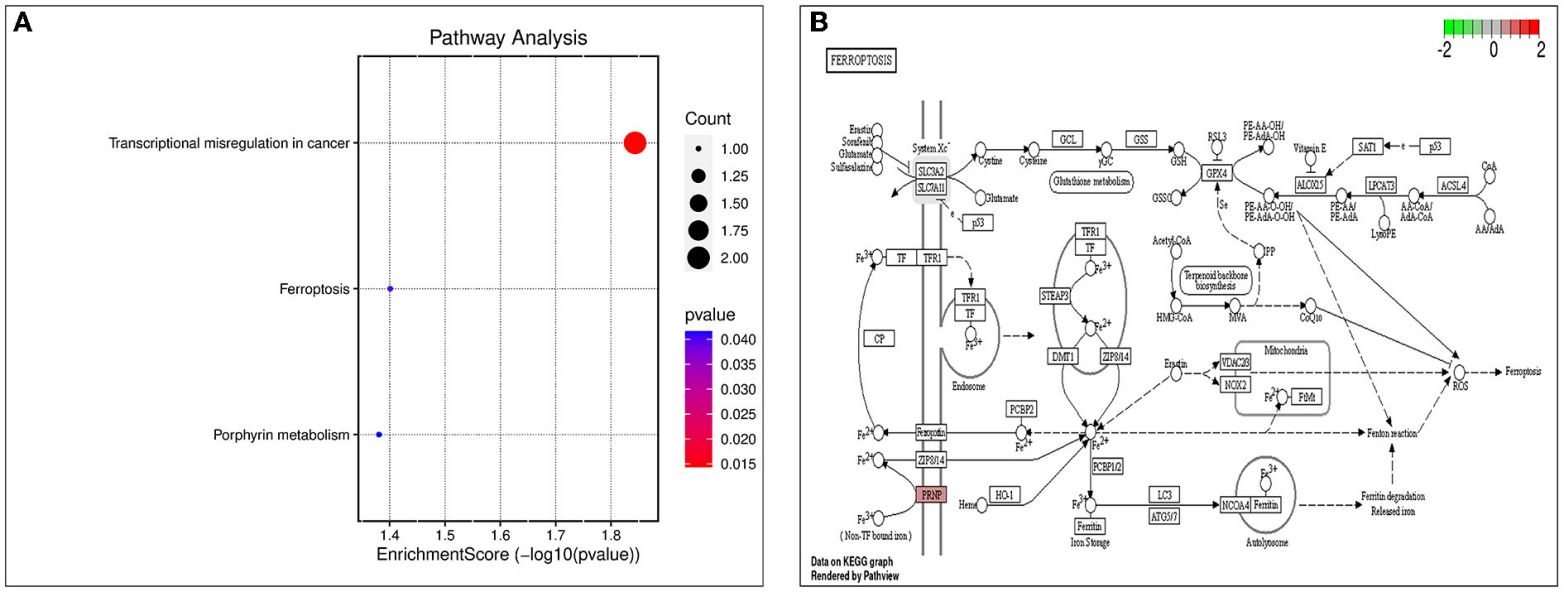
Results of KEGG enrichment analysis. **(A)** KEGG enrichment analysis bubble chart; **(B)** ferroptosis signaling pathway.

The biological processes (BP) associated with the 21 AD-OP-related DEGs were regulation of calcium ion import, endothelial cell proliferation, and inositol phosphate-mediated signaling (Figure 4A). The related cell compositions (CC) mainly included BLOC-1 complex, eukaryotic 48S preinitiation complex, and eukaryotic translation initiation factor 3 complex (Figure 4B). The related molecular functions (MF) mainly enriched were glutamate receptor binding, superoxide-generating NADPH oxidase activator, and G protein-coupled glutamate receptor binding (Figure 4C). Figure 4D shows the GO enrichment features. KEGG pathway enrichment analysis shows that pathways like transcriptional dysregulation in cancer, ferroptosis, porphyrin metabolism, and other immune-related signaling pathways (Figure 5A) were associated with 21 AD-OP-related DEGs in peripheral blood. Furthermore, AD-OP-related DEGs in peripheral blood function were closely related to the ferroptosis signaling pathway (Figure 5B).

### Establishment of OP-inflammatory response-related gene expression matrix

**Figure 6:**
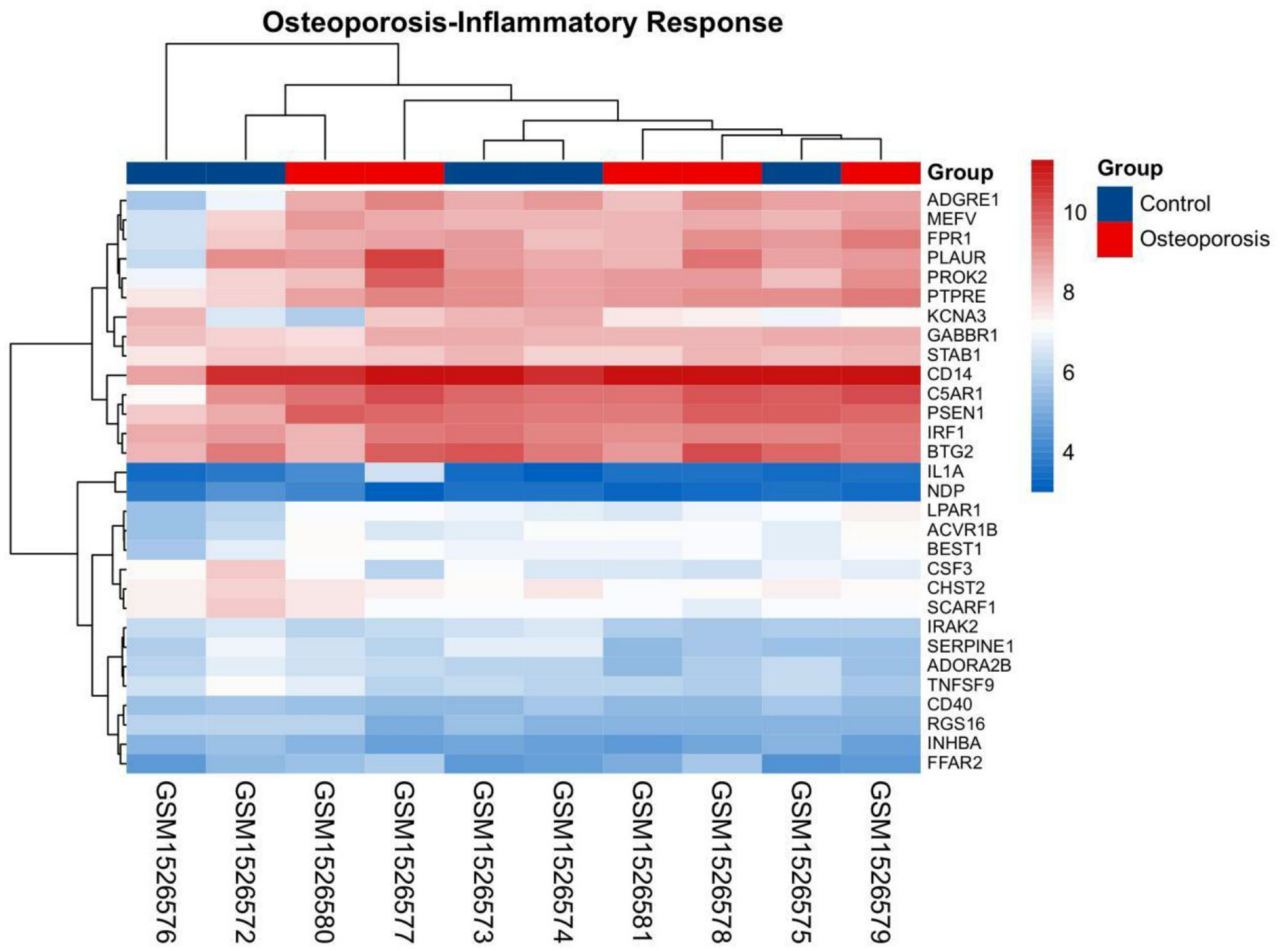
Heat map for clustering of inflammatory response-related genes in OP.

The gene set related to the inflammatory response was downloaded from GSEA. The OP-related GSE62402 dataset was retrieved from GEO based on the pre-set filters. R software was used to organize and analyze the metabolomics-related expression matrix. According to the screening criteria set earlier, two differentially expressed mRNAs were identified, *PROK2* was upregulated, and *CSF3* was downregulated. Figure 6 shows a heat map of the OP-inflammatory response-related gene expression matrix.

### Relative expression of the core target genes associated with OP inflammation

**Figure 7:**
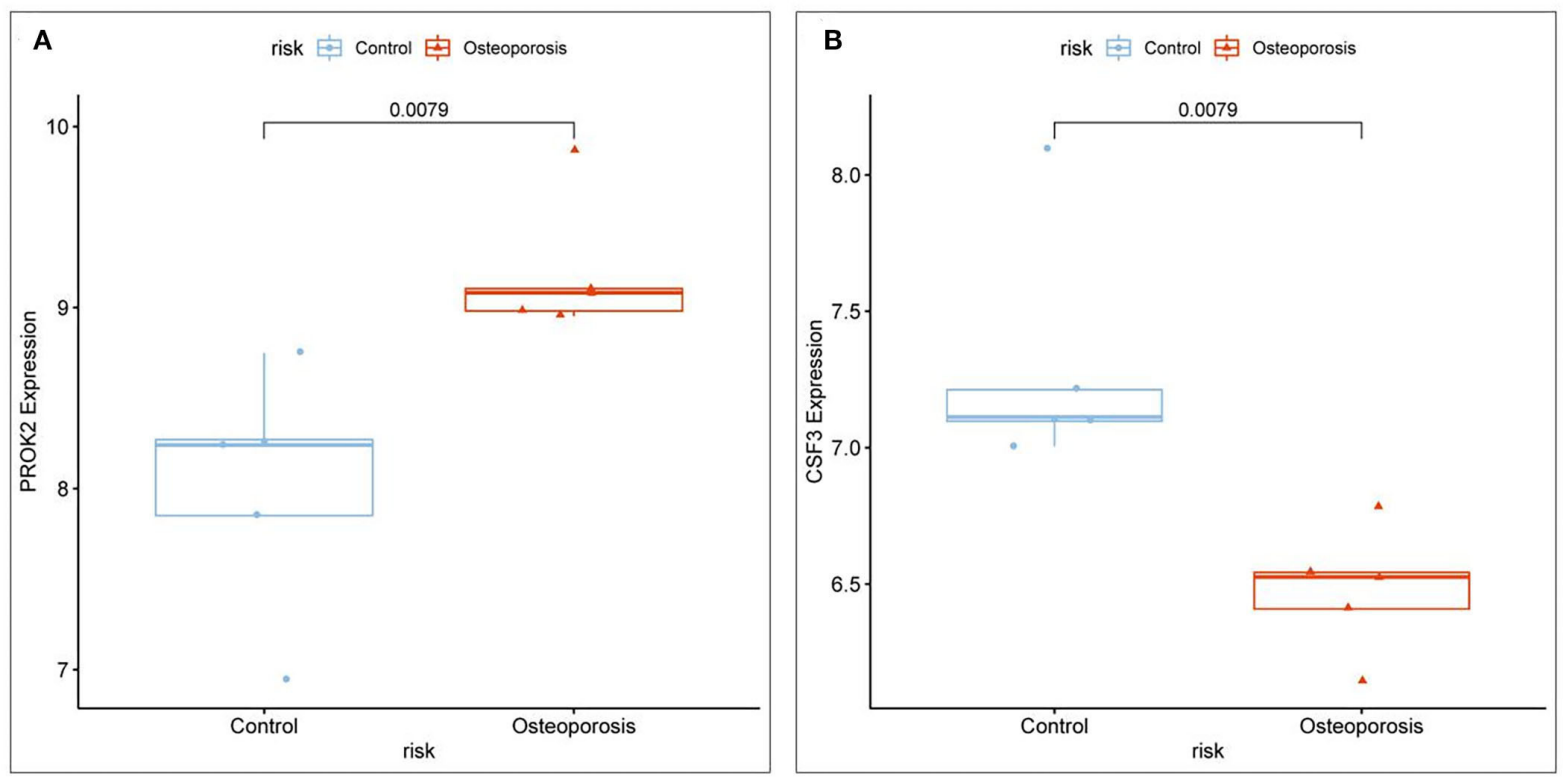
Relative expression levels of core targets involved in OP inflammation. **(A)** *PROK2* relative expression; **(B)** *CSF3* relative expression.

The core OP-inflammation-related genes *PROK2* and *CSF3* were obtained by comprehensive differential expression analysis. The relative expression of *PROK2* and *CSF3* in OP was analyzed. The relevant expression profile of *PROK2* and *CSF3* in OP patients was downloaded from GEO, analyzed by the ggpubr package, and visualized using the box expression map (Figures 7A,B). The results showed that *PROK2* was highly expressed in peripheral blood OP patients compared to normal healthy individuals and the difference was statistically significant (*P* < 0.01). Furthermore, compared to normal healthy individuals, there was a significant reduction in *CSF3* expression in peripheral blood of OP patients (*P* < 0.01).

### GO and KEGG enrichment analysis results of OP-inflammation-related genes

**Figure 8:**
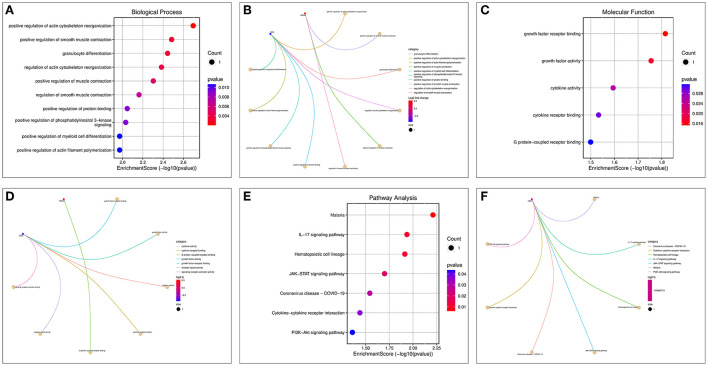
GO and KEGG enrichment analysis of OP-inflammation-related genes. **(A)** BP enrichment bubble diagram; **(B)** BP enrichment arc diagram; **(C)** MF enrichment bubble diagram; **(D)** MF enrichment arc diagram; **(E)** KEGG enrichment bubble diagram; **(F)** KEGG enrichment arc diagram.

Enrichment analysis of the two OP-inflammatory response-related genes enriched BP, such as the regulation of actin cytoskeleton reorganization, smooth muscle contraction, and granulocyte differentiation. Their molecular functions enriched were growth factor receptor binding, growth factor activity, and cytokine activity (Figures 8A–D). The KEGG pathway enrichment analysis found that their functions are mainly associated with malaria, IL-17 signaling pathway, hematopoietic cell lineage, JAK-STAT signaling pathway, and COVID-19 (Figures 8E,F).

### Molecular docking of inflammatory proteins

**Figure 9:**
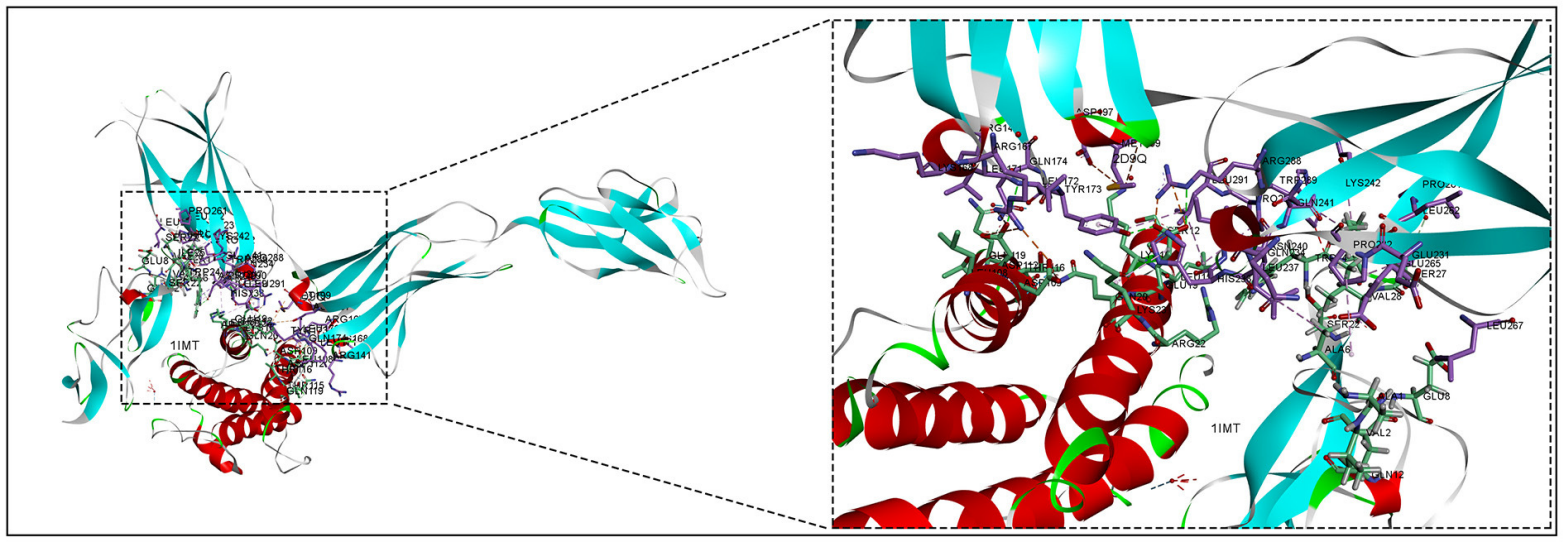
Molecular docking of inflammatory proteins and protein docking model.

The 3D structures of the 1IMT structural domain of *PROK2* protein and the 2D9Q structural domain of *CSF3* protein were downloaded from the PDB database and exported in PDB format. The ZDOCK module of Discovery Studio 2019 software was used to rigidly dock *PROK2* protein to *CSF3* protein. The ZDOCK Score values and their best pose interaction were calculated, as shown in Table 1. The ZDOCK Score of the 1IMT domain of *PROK2* protein and the 2D9Q docking model of *CSF3* protein was −85.085. The 1IMT domain of *PROK2* proteins forms hydrogen bond links with amino acid sites such as B:ARG167:NH1—A:ASP109:OD1, B:ARG167:NH2—A:ASP112:OD1, B:ARG288:NH2—A:GLU19:OE1, A:HOH177:O—A:PRO65:O, and other amino acid sites, whereas A:LYS16:NZ—B:ASP197:O D1, A:LYS16:NZ—B:ASP200:OD1, B:ARG167:NH1—A:ASP112:OD2, B:ARG288:NH1—A:GLU19:OE2, A:LEU15—B:LEU291, and other amino acid sites form electrostatic interactions and water transport bonds. Comprehensive analysis revealed that proteins *PROK2* and *CSF3* formed a stable protein docking model. Figure 9 demonstrates two-dimensional molecular docking constructed using Discovery Studio 2019 software.

**Table 1:** Results of molecular docking vina, discovery studio 2019.

**Receptor**	**Ligand**	**ZDOCK score**	**Hydrogen bond interaction**	**Electrostatic interaction**
*PROK2* (1IMT)	*CSF3* (2D9Q)	−85.085	B:ARG167:NH1—A:ASP109:OD1, B:ARG167:NH2—A:ASP112:OD1, B:ARG288:NH2—A:GLU19:OE1, A:HOH177:O—A:PRO65:O…	A:LYS16:NZ—B:ASP197:OD1, A:LYS16:NZ—B:ASP200:OD1, B:ARG167:NH1—A:ASP112:OD2, B:ARG288:NH1—A:GLU19:OE2, A:LEU15—B:LEU291

## Discussion

Gene expression microarray datasets on Alzheimer's disease and osteoporosis patients' peripheral blood samples were retrieved from the GEO database. The results revealed that two dysregulated proteins, *PROK2* and *CSF3*, were associated with the occurrence of OP in patients with AD. Using rigid protein–protein docking by ZDOCK confirmed that the two proteins form a stable protein docking model, suggesting that the interaction between the two proteins plays an important role in the occurrence of OP in patients with AD.

Prokineticin 2 (*PROK2*) is expressed throughout the central nervous system (35). As a new family of chemokine-like molecules, they are involved in various physiological and pathological processes, including nerve and blood vessel regeneration, pain, inflammation, and neuroinflammation (36–38). A study confirmed that *PROK2* mediates harmful brain injuries (39). In AD, *PROK2* maintains a state of neuroinflammation and causes neurotoxicity (35). Studies show the involvement of *PROK2* in Aβ-induced toxicity, as Aβ peptides increase *PROK2* expression in AD, representing a new class of pathological markers in AD animal models (40–42). Consistent with the previous studies, our results show that *PROK2* expression was upregulated in patients with AD. Furthermore, the *PROK2* expression was not only associated with inflammatory responses in the blood samples of patients with AD but also was a core gene associated with inflammation in OP.

Few studies have shown the *PROK2* expression and functions in OP. Interestingly, previous studies have demonstrated the involvement of *PROK2* in Aβ-mediated toxicity and have a positive correlation with Aβ peptides. It also alters the bone mineral density, which may affect the bone formation and resorption balance, leading to the development of OP (16, 42). Prokineticin receptor 2 (*PROKR2*) is the *PROK2* and G protein-coupled receptor (GPCR). In addition, GPCRs affect bone metabolism by influencing the cytokines and signaling pathways that regulate osteoblasts (OB) and osteoclasts (OC) (43). In addition, *PROK2* is also closely associated with gastrointestinal (GI) function and GI diseases (44). Previous studies have also shown that the OP incidences were significantly higher in patients with GI diseases (45, 46). Hence, we hypothesized that *PROK2* plays an important role in the development of OP. In this study, we show for the first time the upregulation of *PROK2* expression in OP. Furthermore, *PROK2* was a core gene associated with OP inflammation and a common gene differentially expressed between AD and OP patients. Hence, *PROK2* could be potentially used as a molecular marker for predicting the occurrence of OP in patients with AD.

*CSF3* is a member of the colony-stimulating factor family. Together with its receptor *CSF3R, CSF3* is involved in regulating sarcomere cell production, neutrophil function, etc. (47). A study has reported that neutrophil/lymphocyte ratio could be used in predicting the occurrence of OP (48). Zhang et al. demonstrated the expression of RANKL, the osteoclast differentiation factor on the surface of neutrophils. RANKL binds to the osteoclast differentiation factor receptor, RANK, which mediates osteoclast differentiation, thereby enhancing the osteoclast activity. This disrupts bone metabolism, which reduces bone mass (49). However, the relationship between *CSF3* and OP has not been established.

In this study, the expression of *CSF3* was downregulated in OP as a core gene related to OP inflammation. Bone resorption is enhanced during the chronic inflammatory response, reducing the bone formation and promoting OP (50). Previous studies show that *CSF3* is an anti-inflammatory cytokine that clears bacterial pathogens and modulates the inflammatory response (51). Therefore, we postulate the involvement of *CSF3* in inflammation-related biological processes in the progression of OP.

The core inflammation-related genes *PROK2* and *CSF3* involved in OP, identified in this study, were associated with the signal transducer and activator of the transcription (STAT) pathway. The STAT pathway induces astrocyte proliferation and is activated in AD animal models and humans. Previous studies have demonstrated that reactive astrocyte proliferation is a hallmark of the AD signaling pathway (52). STAT3 induces astrocyte proliferation and is activated in human AD and animal models, and reactive astrocyte proliferation is a hallmark of AD (52). During acute inflammation and septic inflammatory conditions, *CSF3* mediates STAT3-dependent upregulation of neutrophil IL-4R (53). Interestingly, an increase in *STAT3* phosphorylation was observed in cells stably expressing PROKR2, which is the receptor for *PROK2* (54). In addition, the STAT signaling pathway plays an important role in the pathogenesis of OP and AD. Inhibiting *STAT3* phosphorylation attenuates learning and causes memory deficits in AD animal models (55). Furthermore, the STAT3 signaling pathway is involved in the progression of OP by altering osteoblast bone metabolism (43, 56). Consistent with the previous studies, the KEGG pathway enrichment analysis revealed that both genes enriched the JAK-STAT signaling pathway and pathways associated with malaria, IL-17 signaling pathway, hematopoietic cell lineage, and COVID-19. GeneCards database (https://ga.genecards.org/#results) shows that both *PROK2* and *CSF3* were associated with the extracellular region. Furthermore, GO analysis revealed the involvement of *PROK2* and *CSF3* in protein binding and their association with VEGF. In this study, we identified the combined role of *PROK2* and *CSF3* in the pathogenesis of AD and OP. Our results reveal those alterations in the inflammatory response pathway in the peripheral blood of patients with AD may affect the occurrence and progression of OP. The docking results show that proteins *PROK2* and *CSF3* could form a stable protein docking model, thus confirming the previous bioinformatics results that the interaction between the *PROK2* and *CSF3* could be involved in the inflammatory-related response to OP in patients with AD.

In this study, using bioinformatics analysis, we demonstrated that the proteins *PROK2* and *CSF3* may be involved in inflammation-related processes in the development of OP in patients with AD and confirmed stable protein interactions between them by docking, thereby verifying the reliability of predictions made by bioinformatics analysis. However, the study has a few shortcomings. The primary technique used in this study was bioinformatics analysis. Hence, further experiments validating the interaction between *PROK2* and *CSF3* proteins are required. The results of our study predict the role of *PROK2* and *CSF3* protein binding in the pathogenesis of OP in patients with AD. However, the mechanism is still unclear and needs further exploration using appropriate experiments.

## Conclusion

AD-related OP may be caused by the interaction between *PROK2* and *CSF3*, two proteins related to OP inflammation. Accordingly, abnormalities/alterations in the inflammatory response in the peripheral blood of patients with AD could influence the progression of OP. Further exploration of targets for treating OP in patients with AD will be facilitated by our study.

## Data availability statement

Publicly available datasets were analyzed in this study. This data can be found at: The gene micro-array data related to Alzheimer's disease and osteoporosis were downloaded from the GEO (gene expression omnibus) database (https://www.ncbi.nlm.nih.gov/geo/): GSE97760, GSE168813, and GSE62402 dataset.

## Author contributions

AW, WZ, YZ, and NH: conceptualization, methodology, software, data curation, writing the original draft preparation, and validation. AW and WZ: visualization, investigation, writing, reviewing, and editing. AW: supervision. All authors contributed to the article and approved the submitted version.

## Conflict of interest

The authors declare that the research was conducted in the absence of any commercial or financial relationships that could be construed as a potential conflict of interest.

## Publisher's note

All claims expressed in this article are solely those of the authors and do not necessarily represent those of their affiliated organizations, or those of the publisher, the editors and the reviewers. Any product that may be evaluated in this article, or claim that may be made by its manufacturer, is not guaranteed or endorsed by the publisher.

## Abbreviations

AD: Alzheimer's disease
OP: Osteoporosis
PPI: Protein–protein interaction
GO: Gene Ontology
KEGG: Kyoto Encyclopedia of Genes and Genomes
DEGs: Differentially expressed genes.
